# Who Are They?

**Published:** 1880-07

**Authors:** 


WHO ARE THEY? It is not our purpose nor wish to *force* our journal *upon* ANY *one*, as seems to have been thought *by a few* persons to whom it has been regularly mailed. A very large number among the most intelligent and respectable members of the profession have cheerfully remitted the amount of annual subscription after receiving a specimen copy or several numbers, and have expressed their approval of the tone and value of the work, also thanking us for giving them this opportunity to subscribe.

There are a few to whom we have been sending the journal and have not yet heard from. We *sincerely hope* that they will *now* find it convenient and pleasant to remit the $2.00, or at least, be kind enough to let us hear from them by letter or postal card.

While thanking our friends for their kind wishes and *substantial* aid, we request them to induce all within their circle who may wish for an independent journal, to subscribe for "*The Independent Practitioner.*"

